# Socioeconomic determinants of mental health outcomes among Hawaii adults

**DOI:** 10.3389/fpubh.2025.1526687

**Published:** 2025-02-24

**Authors:** Ruben Juarez, Binh Le, Daniela Bond-Smith, Carl Bonham, Lisa Sanchez-Johnsen, Alika K. Maunakea

**Affiliations:** ^1^University of Hawaii Economic Research Organization, University of Hawaii at Manoa, Honolulu, HI, United States; ^2^Department of Economics, University of Hawaii at Manoa, Honolulu, HI, United States; ^3^Institute for Health and Equity, Milwaukee, WI, United States; ^4^Department of Psychiatry and Behavioral Medicine, Medical College of Wisconsin, Milwaukee, WI, United States; ^5^Department of Psychiatry, John A. Burns School of Medicine, Honolulu, HI, United States; ^6^Department of Anatomy, Biochemistry and Physiology, John A. Burns School of Medicine, Honolulu, HI, United States

**Keywords:** mental health, food insecurity, depression, suicidal ideation, community safety, employment, Hawaii

## Abstract

**Background:**

Socioeconomic factors play a critical role in influencing mental health outcomes, particularly during periods of crisis such as the COVID-19 pandemic. In Hawaiʻi, working adults face unique challenges related to employment, food security, and trust in community safety measures, which may exacerbate risks for depression, low self-esteem, and suicidal ideation. Understanding the interplay of these factors is crucial to addressing mental health disparities and informing targeted policy interventions.

**Methods:**

This study analyzed data from 2,270 adults aged 18 to 65 residing in Hawaiʻi, collected in 2022. Using probit regression models and conditional inference decision trees, the study assessed the impact of 15 socioeconomic and demographic factors on mental health outcomes, specifically symptoms of depression, low self-esteem, and suicidal ideation. Key variables of interest included food security status, employment, marital status, pre-existing health conditions, and perceptions of COVID-19-related community safety.

**Results:**

The findings revealed significant mental health challenges among the participants, with 39.6% reporting symptoms of depression, 14.7% experiencing low self-esteem, and 4.2% expressing suicidal ideation. Food insecurity emerged as the most significant predictor of poor mental health, particularly for depression and suicidal ideation. Within the food-insecure group, individuals with pre-existing health conditions faced worsened mental health outcomes, while marital status served as a protective factor. Employment reduced the likelihood of depression by 2.8%, and perceptions of community safety during the COVID-19 pandemic were associated with a 9.9% reduction in depression risk.

**Conclusion:**

Food insecurity, particularly when coupled with pre-existing health vulnerabilities, is a critical risk factor for adverse mental health outcomes among working adults in Hawaiʻi. Employment and positive perceptions of community safety were identified as key protective factors. These findings highlight the urgent need for targeted interventions to improve food security and foster community trust and safety.

## 1 Introduction

Mental health is a significant public health concern in the US, with clinical depression affecting over 18 million adults (or one in ten) in a given year, making it the leading cause of disability for young and middle-aged adults aged 15–44 (1). The relationship between depression, self-esteem, and suicide is complex, with depression negatively impacting self-esteem and low self-esteem contributing to the onset or worsening of depression, potentially leading to an increased risk of suicide. Approximately one person dies by suicide every 11 min in the US, with over 48,000 people dying each year due to the effects of depression (2). In addition to these statistics, the COVID-19 pandemic has had a significant impact on mental health, leading to increased stress, anxiety, depression, and other conditions, and these statistics are likely underreported (3).

This study is grounded in a theoretical framework that examines the intersection of social determinants and mental health outcomes. The World Health Organization recognizes socioeconomic factors—categorized as “protective” or “risk” factors—as central to mental health. Protective factors include strong social support networks, job security, healthcare access, and healthy lifestyle choices. Conversely, risk factors such as unemployment, low income, social isolation, and food insecurity significantly contribute to poor mental health outcomes (4).

Several key studies inform this framework. For instance, the most prominent domains of social determinants of mental health as “employment,” “social support,” and “education,” were highlighted (4). More than half of the reviewed studies emphasize “financial hardship,” “housing,” and “demographics” as the social determinants of mental health. On the other hand, “safety/violence,” “housing,” “food insecurity,” and “employment” were identified as critical domains frequently correlated with mental health outcomes (5). Notably, a conceptual framework connecting social and cultural determinants of mental disorders to the Sustainable Development Goals (SDGs) was presented (6). This framework aligns closely with our study as it examines how SDG domains relate to the WHO Commission on the Social Determinants of Health, emphasizing the interplay between structural inequalities and mental health. The Commission on the Social Determinants of Health framework systematically incorporates three key components-the socio-political context, structural determinants and socioeconomic position, and intermediary determinants to identify the social determinants of health inequalities. The framework also shows how major determinants relate to each other and clarify the mechanisms by which social determinants generate the inequality in health and wellbeing (7).

In this study, we investigated for the first time the social determinants and modifiers of mental health among Hawaiʻi adult residents at the end of the COVID-19 pandemic, drawing on a large dataset collected in 2022 of over 2,000 individuals. We examined how personal and demographic characteristics, socioeconomic status, chronic health conditions, trust in information sources (8–10), and perceptions of community safety influenced depression, low self-esteem, and suicidal ideation, while accounting for differences across sex, employment, and racial backgrounds.

This work uniquely addresses a gap in mental health research specific to Hawaiʻi, where limited literature has used data from the 2008 and 2010 Hawaii Behavioral Risk Factor Surveillance System to study depression among Asian and Pacific Islander adults (11, 12). Our study significantly adds to the understanding of interactions between previously unaddressed protective and risk factors, such as food insecurity, trust in various information sources, community safety perceptions, and health conditions related to COVID-19 including long-COVID on mental health, specifically depression, suicidal ideation, and self-esteem. Findings from this study aim to guide targeted interventions and preventive strategies, particularly for vulnerable and minority populations.

## 2 Materials and methods

### 2.1 Data collection

This study utilizes the infrastructure developed in partnership with the State of Hawaiʻi to gather data from 2,200 adult Hawaiʻi residents in May 2022 (baseline) and 1,630 participants in November 2022 (follow-up survey). The research focused on adult individuals of working-age between 18 and 65 years old. We removed individuals with incomplete questionnaire responses, resulting in a sample size of 2,270 observations to 1,430 unique respondents over the two survey periods. The surveys contained over 100 questions covering demographics, employment status, vaccination status, attitudes toward vaccination, mental health, and food insecurity among other data.

### 2.2 Ethics statement

The study was approved by the University of Hawaii Institutional Review Board under study number 2021–00989, and all participants gave informed consent. All methods were performed in accordance with relevant guidelines and regulations.

### 2.3 Metrics and variable criteria

In this study, measures of socioeconomic determinants included demographic characteristics, education level, employment status, household income per person, and food insecurity. Demographic characteristics included race, age, sex, and civil status. We also included individual health outcomes and behaviors such as smoking and drinking, pre-existing health conditions, safety perception, and historical lingering effects from COVID-19. These metrics and those used in the empirical model are shown in Supplementary Table S1.

The metrics used in this study and the variables included in the empirical model are detailed in Supplementary Table S1. These metrics include the specific cut-off points used to define the mental health outcomes—depression, low self-esteem, and suicidal ideation—as binary variables. Each mental health symptom was coded as a binary variable, with a value of 1 indicating the presence of the symptom and 0 indicating its absence. The criteria for these binary classifications were established based on validated scales widely used in public health research. For example:

Depression was assessed using the Center for Epidemiological Studies Depression (CES-D) 10 item scale, with a score of greater than 10 indicating the presence and high risk of depressive symptoms and coded as 1. This threshold is consistent with clinical guidelines for identifying individuals at risk of depression (13–16).

Low self-esteem was measured using the Rosenberg Self-Esteem Scale (17, 18), with a cut-off score of 15. The individuals are classified as low self-esteem with the score of 15 or less, and high or normal self-esteem with the score of above 15. The threshold is based on prior studies that validated its sensitivity and specificity for detecting low self-esteem (19, 20).

Suicidal ideation was identified through a single-item question validated in previous research, with affirmative responses coded as 1. This standardized question has been used in surveys by the Centers for Disease Control and Prevention, including the “Adolescent Behaviors and Experiences Survey” (21) and the “Youth Risk Behavior Survey” (22).

These thresholds were selected to align with established clinical and research standards in mental health assessment. By using these criteria, we ensured that the binary representation of mental health outcomes accurately reflects meaningful distinctions between affected and non-affected individuals.

Furthermore, all predictor variables in the regression models, such as food insecurity, employment status, and community safety perceptions, were coded based on established definitions and thresholds from the literature (23). For instance, food insecurity was categorized using the Department of Agriculture’s 6 Item Food Security Module, which produce a score between 0 and 6 (24, 25). A score of 3 or above indicates the marginal, low, or very low food security and was coded as 1.

This rigorous approach to variable definition and coding was informed by peer-reviewed literature to ensure comparability with existing research and to provide robust, clinically relevant insights into the socioeconomic determinants of mental health.

### 2.4 Methodology

This study evaluated three mental health symptoms: depression, low self-esteem, and suicidal ideation, represented as binary variables with 1 indicating presence and 0 indicating absence of each symptom. We employed parametric and non-parametric methods to analyze the impact of various social determinants and their correlations with mental health symptoms.

First, we used a probit model as the parametric method, specified by Equation 1:


(1) $$Y_{\text{ikjt}}=\beta X_{\text{it}}+\eta_{k}+\rho_{j}+\omega_{t}+\in_{\text{ikjt}}$$

where Y_ikjt_ represents the mental health symptom(s) of individual *i*, working in industry *k* and residing in county *j* at round *t*. The vector X_it_ includes individual-level characteristics, such as employment status, race, age, sex, civil status, long-COVID status, trust in official and unofficial information, community safety perceptions, and household factors (e.g., household size, income, food security). In addition, we controlled for fixed effects for the industry in which individuals work (*η*_k_), the county of residence (*ρ*_j_), and the time of data collection (*ω*_t_) (i.e., spring or fall 2022); the error term is *ε*_ikjt_.

For robustness, we also applied a non-parametric approach—the conditional inference decision tree. This method visually partitions mental health conditions based on predictors by testing for independence between predictors and symptoms. The method is built by performing a significant test on the independence between the predictors and symptoms (26). In order to use the conditional inference decision tree approach, we first used clustering analysis to group the individuals with similar mental health symptoms into homogeneous groups (27). In particular, individuals were clustered based on their mental health profiles: (i) no mental health symptoms, (ii) depression symptoms only, (iii) depression and low self-esteem symptoms together, and (iv) all three symptoms (depression, low self-esteem, suicidal ideation); 96.4% of individuals in this cohort matched one of these four profiles. We then generated a CTREE tree-shaped probability map (R software). This tree diagram, with branches determined by multiplicity-adjusted *p*-values based on Bonferroni’s criterion, illustrates the predictors’ non-linear effects on mental health symptoms (28).

## 3 Results

### 3.1 Descriptive statistics

Participants in this cohort study were recruited from four counties in Hawaiʻi, with the majority (70.9%) residing in Honolulu County, followed by Hawaiʻi County (15.1%), Maui County (8.9%), and Kauai County (4.8%) representative of the working-age state population 18–65 years old. Table 1 provides a summary of participant characteristics.

**Table 1:** Descriptive statistics of the sampled population.

Variable	Mean	SD
Dependent variable		
Depression	0.396	0.489
Low self-esteem	0.147	0.354
Suicidal ideation	0.042	0.201
Independent variable		
Demographic characteristics		
Employment	0.884	0.321
Male	0.337	0.473
Age	45.447	10.958
Married	0.598	0.490
Education	0.736	0.203
Race		
*Caucasian*	0.269	0.443
*Hawaiian and Pacific Islanders*	0.195	0.396
*Asian*	0.444	0.497
*Others*	0.093	0.290
Health conditions		
Long-COVID	0.121	0.326
Pre-existing health condition	0.515	0.500
Alcohol/Tobacco consumption		
Smoking	0.119	0.323
Drinking	0.244	0.430
Household’s characteristics		
Household income per person	1.686	1.183
Food insecurity	0.270	0.444
Trust		
Official trust	0.725	0.223
Unofficial trust	0.575	0.207
Safety against COVID-19	0.613	0.487
	*N* = 2,270	

Most participants (88.4%) were employed. Demographically, 33.7% were male, with an average age of 45.4 years. Nearly 60% were married or living with a domestic partner, and the median education level was a bachelor’s degree. The cohort self-reported predominantly Asian (44.4%), followed by Caucasian (26.9%) and Native Hawaiian and Pacific Islander (19.5%), with 9.3% identifying as “other.”

In terms of health outcomes, 12.1% reported long-COVID, and 51.5% did not report any chronic health conditions. Alcohol consumption was reported by 25, and 12.5% reported smoking. The average household size was four people, with 27% of participants experiencing food insecurity. Trust in information was higher for official sources (average score of 0.72) than for unofficial sources (average score of 0.58), and 61% felt safe or very safe in their community against COVID-19. Self-reported mental health assessments revealed that 39.6% experienced depressive or highly depressive symptoms, 14.7% reported low self-esteem, and 4.2% reported suicidal ideation.

### 3.2 Main results

#### 3.2.1 Employment and mental health

Our results demonstrated a positive association between employment and mental health. These findings are robust to different model specifications that control for industry, survey round, and county fixed effects. Specifically, we found that compared to those who were unemployed, employed individuals were 2.8% less likely to experience depression [−0.028, *p* < 0.05; 95%CI = (−0.052; −0.004)], 6.8% less likely to experience low self-esteem [−0.068, *p* < 0.001; 95%CI = (−0.081; −0.054)], and 2.5% less likely to report suicidal ideation [−0.025, *p* < 0.001; 95%CI = (−0.031; −0.019)]. Supplementary Figure S1 illustrates the difference in depression and self-esteem scores between employed and unemployed individuals, with unemployed individuals having significantly higher scores on depression and lower scores on self-esteem than their employed counterparts.

#### 3.2.2 Sex and mental health

Our study supports previous reports of sex differences in depression. Men were 5.4% less likely to be depressed compared to women [−0.054, *p* < 0.001; 95%CI = (−0.075; −0.034)].

#### 3.2.3 Civil status and mental health

Married individuals had better mental health than unmarried people. Specifically, being married decreased the likelihood of depression by 8.8% [−0.088, *p* < 0.001; 95%CI = (−0.103; −0.072)], low self-esteem by 9.6%[−0.096, *p* < 0.001; 95%CI = (−0.131; −0.060)], and suicidal ideation by 2.9% [−0.029, *p* < 0.001; 95%CI = (−0.041; −0.018)].

#### 3.2.4 Age and mental health

Within the cohort, older individuals were 0.3% [−0.003, *p* < 0.001; 95%CI = (−0.004; −0.002)] less likely to have low self-esteem than younger individuals. Supplementary Figure S2 illustrates the mean scores of depression and self-esteem across different age groups and employment status. In this repeated cross-sectional analysis, we observed decreased levels of depression concomitant with increased self-esteem scores with age. As discussed above, unemployed individuals exhibited higher depression scores compared to employed individuals, while those that were employed tended to report higher self-esteem than those unemployed. Among the unemployed population, a U-shaped pattern of depression scores was evident, with individuals aged less than 29 and those aged 60 or above displaying lower depression scores than other age groups. The largest disparities in depression and self-esteem scores between the employed and unemployed were observed among people aged between 30 and 60 years old.

#### 3.2.5 Race and mental health

Mental health conditions varied by race/ethnic group, with Native Hawaiian and Pacific Islander individuals reporting lower rates of depression by 6.8% [−0.068, *p* < 0.01; 95%CI = (−0.118; −0.017)] and lower rates of low self-esteem by 7.9% [−0.079, *p* < 0.05; 95%CI = (−0.152; −0.006)] compared to other races. Similarly, Asian individuals report lower rates of depression by 8.6% [−0.086, *p* < 0.001; 95%CI = (−0.130; −0.042)] than other race/ethnic groups. Conversely, Caucasians are more likely to experience suicidal ideation by 1.8% [0.018, *p* < 0.001; 95%CI = (0.008; 0.028)].

#### 3.2.6 Long-COVID and mental health

Among those testing positive for COVID-19, 30% reported experiencing long-COVID-19. Individuals who experienced long-COVID were more likely to report mental health issues, with those individuals being 8.7% more likely to experience depression [0.087, *p* < 0.01; 95%CI = (0.020; 0.153)] compared to those without long-COVID.

#### 3.2.7 Chronic health conditions and mental health

Individuals without chronic health conditions were 12.8% less likely to report depressive symptoms [−0.128, *p* < 0.001; 95%CI = (−0.165; −0.091)], low self-esteem by 8.7% [−0.087, *p* < 0.001; 95%CI = (−0.098; −0.076)] and suicidal ideation by 4.8% [−0.048, *p* < 0.001; 95%CI = (−0.059; −0.037)] compared to those with chronic health conditions.

#### 3.2.8 Cigarette Smoking, drinking and mental health

Our findings indicated that alcohol consumption had little impact on mental health, with the exception of depression. Drinking was associated with a 4.3% increase in the probability of depression [0.043, *p* < 0.01; 95%CI = (0.012; 0.073)]. In contrast, smoking was strongly linked to mental health conditions. Compared to non-smokers, smokers had a 5.1% [0.051, *p* < 0.01; 95%CI = (0.019; 0.083)] higher probability of depression, a 5.2% [0.052, *p* < 0.01; 95%CI = (0.018; 0.086)] greater likelihood of low self-esteem, and a 1.8% [0.018, *p* < 0.05; 95%CI = (0.003; 0.032)] higher probability of suicidal ideation.

#### 3.2.9 Food insecurity, household income per person, and mental health

**Figure 1:**
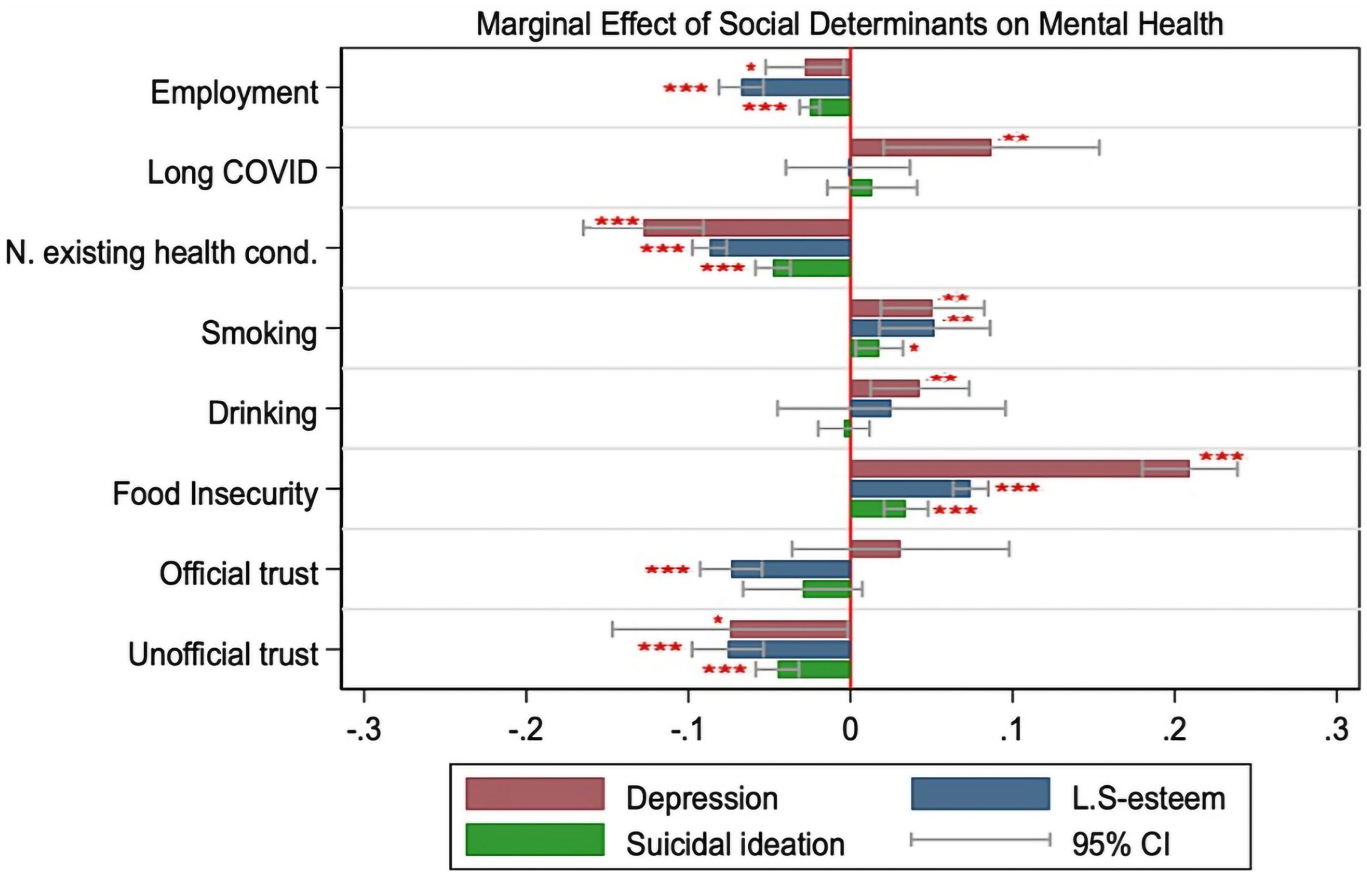
The graphs depict the marginal effect of social determinants on mental health outcomes (Depression, Low Self-esteem and Suicidal ideation). Full results of the regressions are in Supplementary Table S2 (*N* = 2,270). The threshold of 0 is the red vertical lines, the negative marginal effects are the left bars while the positive marginal effects are the right bars of the threshold. Red stars indicate statistical significance at *p < 0.05, **p < 0.01, ***p < 0.001.

Individuals experiencing food insecurity were at a greater risk of experiencing mental health conditions. Specifically, they had a 20.9% [0.209, *p* < 0.001; 95%CI = (0.180; 0.239)] higher likelihood of depressive symptoms, a 7.4% [0.074, *p* < 0.001; 95%CI = (0.063; 0.085)] higher probability of low self-esteem, and a 3.4% [0.034, *p* < 0.001; 95%CI = (0.021; 0.048)] higher likelihood of suicidal ideation. We did not observe a significant statistical association between household income per person and mental health metrics (Figure 1).

#### 3.2.10 Trust and mental health

Regarding the relationship between trust and mental health, we found that both trust in unofficial and trust in official information have an inverse correlation with components of mental health. Specifically, trust in unofficial information was associated with a 7.4% [−0.074, *p* < 0.05; 95%CI = (−0.147; −0.002)] decrease in the probability of having depressive symptoms, and a 4.5% [−0.045, *p* < 0.001; 95%CI = (−0.058; −0.032)] decrease in the probability of reporting suicidal ideation. Trust in official information (i.e., doctors, healthcare providers, news on radio, TV, and governments) was associated with a 7.4% [−0.074, *p* < 0.001; 95%CI = (−0.093; −0.055)] decrease in the probability of low self-esteem.

#### 3.2.11 Neighborhood safety perceptions and mental health

Individuals who feel “safe” and “very safe” in their community were less likely to experience mental health issues. Safety perceptions regarding COVID-19 in the community reduced the probability of depressive symptoms by 9.9% [−0.099, *p* < 0.001; 95%CI = (−0.128; −0.070)].

### 3.3 Robustness check

From clustering of behaviors analysis, we found that three mental health issues were significantly associated with each other, such that having one mental health issue was correlated with increasing the probability of having another mental health issue. In our cohort, 95% of individuals who reported suicidal ideation also experienced low self-esteem and depression Table 2. By using the non-parametric Chi-square test of independence (29), we found that mental health issues were mutually dependent on one another in the baseline sample. People who reported low self-esteem were also more likely to have reported depressive symptoms [$\chi^{2}\left(1\right)=218.76$, *p* = 0.000] and people who reported suicidal ideation were more likely to have reported depressive symptoms [$\chi^{2}\left(1\right)=88.21$, *p* = 0.000]. Individuals who reported having low self-esteem were also more likely to report suicidal ideation [$\chi^{2}\left(1\right)=114.8$, *p* = 0.000].

**Table 2:** Clustering of mental health outcomes and we note that 96.6% of observations fall in categories A-0, B-1, C-2 or D-3 (bold rows), and were used for the non-parametric approach, the conditional inference decision tree.

				Round 1 (*N* = 1,411)		Round 2 (*N* = 859)	
Symptom	Dep	LSE	Suicide	Frequency	Percentage	Frequency	Percentage
**D-3**	**Y**	**Y**	**Y**	**42**	**2.98%**	**14**	**1.63%**
2	Y	N	Y	29	2.06%	5	0.58%
**C-2**	**Y**	**Y**	**N**	**145**	**10.28%**	**88**	**10.24%**
2	N	Y	Y	2	0.14%	0	0.00%
**B-1**	**Y**	**N**	**N**	**373**	**26.44%**	**203**	**23.63%**
1	N	N	Y	3	0.21%	1	0.12%
1	N	Y	N	24	1.70%	19	2.21%
**A-0**	**N**	**N**	**N**	**793**	**56.20%**	**529**	**61.58%**

We further analyzed the association between predictors and mental health conditions in the clustering sample by using a non-parametric approach of conditional inference trees (26). In conditional inference trees, only predictors that are statistically significant with *p*-values greater than 0.05 are displayed. Therefore, this method is informative and better at determining the true effect of the predictors. In particular, from the clustering sample of mental health conditions, we only focused on four dominant groups: A-None of the mental health symptoms (58.24%), B-One symptom (depression) (25.37%), C-Two symptoms (depression and low self-esteem) (10.26%), and D- Three symptoms (depression, low self-esteem, and suicidal ideation) (2.47%).

**Figure 2:**
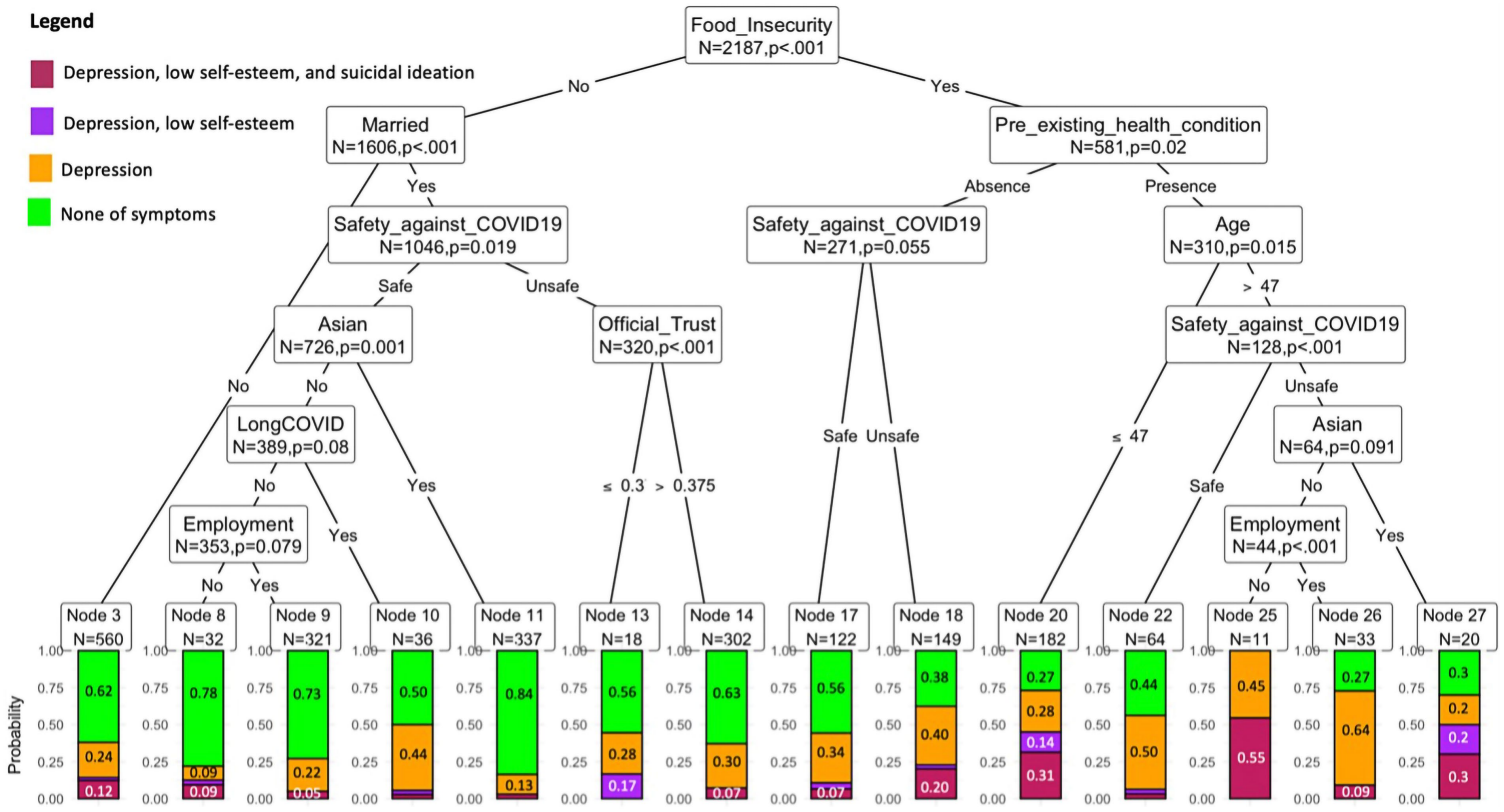
The graph depicts the decision tree for classifying the number of mental health outcomes with a statistical significance level of 5%. Each node provides the information of node size indicated by the number of observations and the probability of mental health symptoms. Only nodes with the variables satisfying the statistical significance level of 5% are included in the tree. The outcomes are described in color categories (green- none of mental health symptoms), orange- one symptom (depression), purple- two symptoms (depression + low self-esteem), red-three symptoms (depression + low self-esteem + suicidal ideation). The y-axis is the frequency (probability) of the mental health outcomes. The determinants and modifiers found in this tree are robust to other statistical significance levels, including 10 and 1% (see more details in Supplementary Figures S3A,B).

The results of conditional inference decision tree analysis are illustrated in Figure 2. Consistent with our probit estimation, participants who reported having food insecurity, pre-existing health conditions, long COVID, feeling unsafe in their community, and being unemployed have a higher probability of reporting mental health issues. Participants who are food insecure are more likely to have mental health issues than food-secure people. Asian people who are food secure, are married or living with their partners, and feel safe in their community are less likely to report mental health symptoms. Eighty-four percent of these people are classified into none of the symptoms group, and 13% of these individuals are in the group of one symptom (node 11). By contrast, non-Asian unemployed people aged 47 years old who are food insecure and have pre-existing health conditions are more likely to have mental health symptoms. Forty-five percent of these individuals are classified in the one-symptom group and 55% of them are in a three-symptom group (node 25). The absence of pre-existing health conditions and feeling safe in the community are considered as protectors of the food-insecure people (node 17).

## 4 Discussion

This study provides the most comprehensive analysis to date of the determinants of mental health—namely, depression, low self-esteem, and suicidal ideation—among adults in Hawaiʻi. By leveraging a large and diverse dataset, the study offers valuable insights into the complex relationships between socioeconomic factors and mental health outcomes. It uniquely addresses the challenges faced by Hawaiʻi’s multicultural population, providing critical information on how food insecurity, employment, race, and chronic health conditions intersect to influence mental wellbeing.

One of the study’s key strengths is its dual methodological approach, combining parametric and non-parametric analysis to uncover linear and non-linear patterns. Additionally, the focus on protective factors, such as employment and community safety perceptions, alongside risk factors like food insecurity, allows for a balanced understanding of mental health vulnerabilities and resilience. The findings are timely, reflecting the post-COVID-19 context, offering actionable insights for policies and interventions to improve mental health outcomes in Hawaiʻi’s unique population. These strengths underscore the study’s importance as a resource for guiding targeted, community-centered strategies to address mental health disparities.

### 4.1 Food insecurity and mental health

Our findings reveal that food insecurity is a primary driver of mental health challenges, with strong associations across depression, low self-esteem, and suicidal ideation. Food insecurity was particularly impactful among unemployed individuals, where it exacerbated mental health disparities. Employment offered a strong protective factor only for those experiencing food insecurity, associated with lower depression rates, higher self-esteem, and reduced suicidal ideation. Additionally, food-insecure individuals reported a heightened negative impact of alcohol consumption on depression and self-esteem, further underscoring the vulnerability of this group. These results suggest that addressing food insecurity could yield substantial mental health benefits across multiple population segments in Hawaiʻi.

### 4.2 Employment and mental health

Our study supports prior research linking employment to improved mental health outcomes (30–36). Employment was associated with lower symptoms of depression, suicidal ideation, and higher self-esteem. Interestingly, employment also modified the effects of other risk factors: employed individuals facing food insecurity or chronic health conditions had fewer mental health symptoms than their unemployed counterparts. The disparities between employed and unemployed adults, especially those aged 30 to 60, are pronounced, suggesting that employment not only offers direct mental health benefits but also buffers against other socioeconomic challenges.

### 4.3 Sex differences in mental health

Significant sex differences emerged in mental health outcomes, with women reporting higher rates of depression but lower instances of suicidal ideation compared to men. This aligns with previous findings that stressors such as family responsibilities, multiple roles, and potential discrimination may contribute to depressive symptoms among women (37). Employment and supportive social networks appeared particularly beneficial for women, correlating with improved mental health and higher self-esteem (38, 39).

### 4.4 Race/ethnicity and mental health

Mental health disparities across racial and ethnic groups reflected complex sociocultural influences (40). Asian participants reported lower depressive symptoms compared to Caucasians, Native Hawaiians, and Pacific Islanders (41). Food insecurity significantly increased depression and suicidal ideation across all racial groups. Notably, trust in official information sources was particularly protective against depression and low self-esteem among Caucasians, suggesting that interventions promoting trustworthy information may help reduce mental health risks in specific demographics.

### 4.5 Civil status and mental health

Consistent with existing literature, our results show that marriage or long-term partnership is associated with improved mental health outcomes, likely due to increased psychological, social, and economic resources (42, 43). This protective effect was evident across both depression and self-esteem metrics, emphasizing the value of stable relationships for mental health resilience (44).

### 4.6 Trust and mental health

Our study examined trust in both official and unofficial information sources as potential mental health determinants using validated instruments for Hawaii’s populations (8–10). Both types of trust correlated with lower rates of depression, higher self-esteem, and decreased suicidal ideation. These findings reinforce the importance of trust as a supportive factor for mental health, suggesting that fostering reliable information channels may be beneficial in alleviating mental health symptoms (45–49).

### 4.7 Long-COVID, safety perception against COVID-19 and mental health

COVID-19-related variables, including long-COVID and perceptions of community safety, were also significant. Individuals with long-COVID reported higher depression and suicidal ideation, consistent with findings on the extended impact of chronic symptoms on mental wellbeing. Conversely, feeling safe in the community was associated with fewer depressive symptoms, higher self-esteem, and lower suicidal ideation. This protective association aligns with research on community safety, indicating that a stable and safe environment mitigates mental health risks, particularly in uncertain times (50, 51).

### 4.8 Limitations

This study has several limitations. First, self-reported survey data may underrepresent depressive symptoms, suicidal ideation, and behaviors like substance use due to social stigma, introducing potential bias. These self-reports differ from clinically-administered diagnoses, which may warrant further study to confirm consistency. Second, the study lacks detailed data on factors like financial hardship, coping strategies, and emotional intelligence that could influence mental health outcomes. Third, the sample size was limited to adults 18–65 and may not represent the State of Hawaii. Finally, although the conditional inference decision tree model has reasonable precision to detect complex associations between social determinants and mental health, some subtle effects would be neglected because of the stopping rules and the pruning procedures. Despite these limitations, this study offers valuable insights into how social determinants impact mental health in Hawaiʻi’s adult population, which may inform the development of targeted interventions to improve mental health outcomes.

## 5 Conclusion

This study highlights the profound influence of socioeconomic factors on mental health, with food insecurity emerging as the most critical risk factor for depression, low self-esteem, and suicidal ideation among working adults in Hawaiʻi. The interplay between food insecurity and pre-existing health conditions exacerbates mental health vulnerabilities, underscoring the urgent need for interventions to alleviate food insecurity and address associated challenges. At the same time, employment and positive perceptions of community safety were identified as key protective factors, suggesting that efforts to enhance job security and foster a sense of safety within communities can play a vital role in improving mental wellbeing.

These findings provide actionable insights for policymakers, healthcare providers, and community organizations aiming to reduce mental health disparities. By addressing food insecurity and bolstering protective factors like employment and community safety perceptions, targeted interventions can promote resilience and improve outcomes for Hawaiʻi’s diverse population. Future research should build on these insights by exploring long-term impacts and tailoring strategies to specific subpopulations, ensuring that solutions are both equitable and sustainable. This study serves as a foundation for creating community-centered policies and programs that address the root causes of mental health challenges and promote holistic wellbeing.

## Data Availability

The raw data supporting the conclusions of this article will be made available by the authors, without undue reservation.

## References

[1] Bernstein CA Hershfield B Cohen DC. Psychiatry in the USA: an overview. Int Psychiatry. (2010) 7:90–2. doi: 10.1192/S1749367600006020 31508054 PMC6734990

[2] Facts About Suicide. Suicide. CDC. (2023). Available at: https://www.cdc.gov/suicide/facts/index.html (Accessed November 16, 2023).

[3] COVID-19 pandemic triggers 25% increase in prevalence of anxiety and depression worldwide. (2023). Available at: https://www.who.int/news/item/02-03-2022-covid-19-pandemic-triggers-25-increase-in-prevalence-of-anxiety-and-depression-worldwide (Accessed November 16, 2023). PMC9998057 35414629

[4] Handerer F Kinderman P Shafti M Tai S. A scoping review and narrative synthesis comparing the constructs of social determinants of health and social determinants of mental health: Matryoshka or two independent constructs? Front Psychiatry. (2022) 13:848556. doi: 10.3389/fpsyt.2022.848556 35492698 PMC9046700

[5] Moen M Storr C German D Friedmann E Johantgen M. A review of tools to screen for social determinants of health in the United States: a practice brief. Popul Health Manag. (2020) 23:422–9. doi: 10.1089/pop.2019.0158 PMID: 31910355, PMID: 31910355 PMC7864106

[6] Lund C Brooke-Sumner C Baingana F Baron EC Breuer E Chandra P. Social determinants of mental disorders and the sustainable development goals: a systematic review of reviews. Lancet Psychiatry. (2018) 5:357–69. doi: 10.1016/s2215-0366(18)30060-9, PMID: 29580610

[7] World Health Organization. A conceptual framework for action on the social determinants of health. Geneva, Switzerland: The WHO Document Production Services. (2010) 76.

[8] Juarez R Phankitnirundorn K Okihiro M Maunakea AK. Opposing role of trust as a modifier of COVID-19 vaccine uptake in an indigenous population. Vaccine. (2022) 10:968. doi: 10.3390/vaccines10060968, PMID: 35746577 PMC9229995

[9] Juarez R Kang Z Okihiro M Garcia BK Phankitnirundorn K Maunakea AK. Dynamics of trust and consumption of COVID-19 information implicate a mechanism for COVID-19 vaccine and booster uptake. Vaccine. (2022) 10:1435. doi: 10.3390/vaccines10091435, PMID: 36146513 PMC9506487

[10] Juarez R Phankitnirundorn K Ramirez A Peres R Maunakea AK Okihiro M. Vaccine-associated shifts in SARS-CoV-2 infectivity among the native Hawaiian and other Pacific islander population in Hawaii. Am J Public Health. (2022) 112:S896–9. doi: 10.2105/AJPH.2022.306973, PMID: 36108254 PMC9707710

[11] Aczon-Armstrong M Inouye J Reyes-Salvail F. Depression and chronic illness: Asian/Pacific islander adults in Hawaii. Issues Ment Health Nurs. 34:169–79. doi: 10.3109/01612840.2012.738356 23477437

[12] McGee RE Thompson NJ. Unemployment and depression among emerging adults in 12 states, behavioral risk factor surveillance system, 2010. Prev Chronic Dis. (2015) 12:140451:E38. doi: 10.5888/pcd12.140451, PMID: 25789499 PMC4372159

[13] Siddaway AP Wood AM Taylor PJ. The Center for Epidemiologic Studies-Depression (CES-D) scale measures a continuum from well-being to depression: testing two key predictions of positive clinical psychology. J Affect Disord. (2017) 213:180–6. doi: 10.1016/j.jad.2017.02.015, PMID: 28254608 PMC6191531

[14] Turvey CL Wallace RB Herzog R. A revised CES-D measure of depressive symptoms and a DSM-based measure of major depressive episodes in the elderly. Int Psychogeriatr. (1999) 11:139–48. doi: 10.1017/S1041610299005694, PMID: 11475428

[15] Irwin M Artin KH Oxman MN. Screening for depression in the older adult: criterion validity of the 10-item Center for Epidemiological Studies Depression Scale (CES-D). Arch Intern Med. (1999) 159:1701–4. doi: 10.1001/archinte.159.15.1701, PMID: 10448771

[16] Zhang W O’Brien N Forrest JI Salters KA Patterson TL Montaner JSG. Validating a shortened depression scale (10 item CES-D) among HIV-positive people in British Columbia, Canada. PLoS One. (2012) 7:e40793. doi: 10.1371/journal.pone.0040793, PMID: 22829885 PMC3400644

[17] Rosenberg M. Rosenberg self-esteem scale (RSE). Acceptance and commitment therapy/Measures package. Princeton University Press. (1965).

[18] Rosenberg M. Conceiving the self. New York: Basic Books. (1979).

[19] Heatherton TF Wyland CL. Assessing self-esteem In: Positive psychological assessment: a handbook of models and measures. Washington, DC: American Psychological Association (2003). 219–33.

[20] Andrews B Brown GW. Self-esteem and vulnerability to depression: The concurrent validity of interview and questionnaire measures. J Abnorm Psychol. (1993) 102:565–72. doi: 10.1037//0021-843X.102.4.565 8282925

[21] Jones SE. Mental health, suicidality, and connectedness among high school students during the COVID-19 pandemic — adolescent behaviors and experiences survey, United States, January–June 2021. MMWR Suppl. (2022) 71:16–21. doi: 10.15585/mmwr.su7103a3 35358165 PMC8979602

[22] Ivey-Stephenson AZ. Suicidal ideation and behaviors among high school students — youth risk behavior survey, United States. MMWR Suppl. (2019) 69:47–55. doi: 10.15585/mmwr.su6901a6, PMID: 32817610 PMC7440198

[23] Jones AD. Food insecurity and mental health status: a global analysis of 149 countries. Am J Prev Med. (2017) 53:264–73. doi: 10.1016/j.amepre.2017.04.008, PMID: 28457747

[24] Blumberg SJ Bialostosky K Hamilton WL Briefel RR. The effectiveness of a short form of the household food security scale. Am J Public Health. (1999) 89:1231–4. doi: 10.2105/AJPH.89.8.1231, PMID: 10432912 PMC1508674

[25] Keenan DP Olson C Hersey JC Parmer SM. Measures of food insecurity/security. J Nutr Educ. (2001) 33:S49–58. doi: 10.1016/S1499-4046(06)60069-9, PMID: 12857544

[26] Hothorn T Hornik K Zeileis A. Ctree: conditional inference trees. The comprehensive R archive network.

[27] Liao M Li Y Kianifard F Obi E Arcona S. Cluster analysis and its application to healthcare claims data: a study of end-stage renal disease patients who initiated hemodialysis. BMC Nephrol. (2016) 17:25. doi: 10.1186/s12882-016-0238-2, PMID: 26936756 PMC4776444

[28] Noh HG Song MS Park SH. An unbiased method for constructing multilabel classification trees. Comput Stat Data Anal. (2004) 47:149–64. doi: 10.1016/j.csda.2003.10.009

[29] McHugh ML. The chi-square test of independence. Biochem Med. (2013) 23:143–9. doi: 10.11613/BM.2013.018, PMID: 23894860 PMC3900058

[30] Disney L. The impact of employment on immigrant mental health: results from a National Survey. Soc Work. (2021) 66:93–100. doi: 10.1093/sw/swab005, PMID: 33842958

[31] Ruffolo M Price D Schoultz M Leung J Bonsaksen T Thygesen H. Employment uncertainty and mental health during the COVID-19 pandemic initial social distancing implementation: a cross-national study. Glob Soc Welf. (2021) 8:141–50. doi: 10.1007/s40609-020-00201-4, PMID: 33432284 PMC7788173

[32] Mokona H Yohannes K Ayano G. Youth unemployment and mental health: prevalence and associated factors of depression among unemployed young adults in Gedeo zone, southern Ethiopia. Int J Ment Health Syst. (2020) 14:61. doi: 10.1186/s13033-020-00395-2, PMID: 32782471 PMC7414568

[33] Van Der Noordt M Jzelenberg H Droomers M Proper KI. Health effects of employment: a systematic review of prospective studies. Occup Environ Med. (2014) 71:730–6. doi: 10.1136/oemed-2013-101891, PMID: 24556535

[34] Paul KI Moser K. Unemployment impairs mental health: meta-analyses. J Vocat Behav. (2009) 74:264–82. doi: 10.1016/j.jvb.2009.01.001

[35] Park S Chan KCG Williams EC. Gain of employment and perceived health status among previously unemployed persons: evidence from a longitudinal study in the United States. Public Health. (2016) 133:83–90. doi: 10.1016/j.puhe.2015.11.008, PMID: 26718423

[36] Murphy GC Athanasou JA. The effect of unemployment on mental health. J Occupat Organ Psyc. (1999) 72:83–99. doi: 10.1348/096317999166518

[37] Platt J Prins S Bates L Keyes K. Unequal depression for equal work? How the wage gap explains gendered disparities in mood disorders. Soc Sci Med. (2016) 149:1–8. doi: 10.1016/j.socscimed.2015.11.056 PMID: 26689629, PMID: 26689629 PMC4801117

[38] Harris MA Orth U. The link between self-esteem and social relationships: a meta-analysis of longitudinal studies. J Pers Soc Psychol. (2020) 119:1459–77. doi: 10.1037/pspp0000265 PMID: 31556680, PMID: 31556680

[39] Leupp K. Even supermoms get the blues: employment, gender attitudes, and depression. Soc Ment Health. (2019) 9:316–33. doi: 10.1177/2156869318785406

[40] General US Surgeon. Mental health: culture, race, and ethnicity. A supplement to mental health: a report of the surgeon general US Department of Health and Human Services. (2001). 20669516

[41] Mukherjee S Trepka MJ Pierre-Victor D Bahelah R Avent T. Racial/ethnic disparities in antenatal depression in the United States: a systematic review. Matern Child Health J. (2016) 20:1780–97. doi: 10.1007/s10995-016-1989-x, PMID: 27016352

[42] Uecker JE. Marriage and mental health among young adults. J Health Soc Behav. (2012) 53:67–83. doi: 10.1177/0022146511419206, PMID: 22328171 PMC3390929

[43] Liu CH Zhang E Wong GTF Hyun S Hahm H. “Chris.” factors associated with depression, anxiety, and PTSD symptomatology during the COVID-19 pandemic: clinical implications for U.S. young adult mental health. Psychiatry Res. (2020) 290:113172. doi: 10.1016/j.psychres.2020.113172, PMID: 32512357 PMC7263263

[44] Simon RW Barrett AE. Nonmarital romantic relationships and mental health in early adulthood: does the association differ for women and men? J Health Soc Behav. (2010) 51:168–82. doi: 10.1177/0022146510372343, PMID: 20617757

[45] Clarke A Meredith PJ Rose TA. Interpersonal trust reported by adolescents living with mental illness: a scoping review. Adolescent Res Rev. (2021) 6:165–98. doi: 10.1007/s40894-020-00141-2

[46] Jasielska D. The moderating role of kindness on the relation between trust and happiness. Curr Psychol. (2020) 39:2065–73. doi: 10.1007/s12144-018-9886-7

[47] Nickerson A Liddell BJ Keegan D Edwards B Felmingham KL Forbes D. Longitudinal association between trust, psychological symptoms and community engagement in resettled refugees. Psychol Med. (2019) 49:1661–9. doi: 10.1017/S0033291718002246, PMID: 30160232

[48] Silva M Loureiro A Graça C. Social determinants of mental health: a review of the evidence. Eur J Psychiatry. (2016) 30:259–92.

[49] Graves M Penner F Sharp C. Interpersonal trust in adolescents with psychiatric disorders and borderline pathology. Scand J Child Adolesc Psychiatry Psychol. (2021) 9:176–86. doi: 10.21307/sjcapp-2021-020, PMID: 34805011 PMC8596190

[50] Stansfeld SA Rothon C das-Munshi J Mathews C Adams A Clark C. Exposure to violence and mental health of adolescents: south African health and well-being study. BJPsych Open. (2017) 3:257–64. doi: 10.1192/bjpo.bp.117.004861, PMID: 29093828 PMC5643877

[51] Chen W-Y Corvo K Lee Y Hahm HC. Longitudinal trajectory of adolescent exposure to community violence and depressive symptoms among adolescents and young adults: understanding the effect of mental health service usage. Community Ment Health J. (2017) 53:39–52. doi: 10.1007/s10597-016-0031-5, PMID: 27286840

