# Supplementary material for: Socioeconomic determinants of mental health outcomes among Hawaii adults
Source: Front Public Health. 2025 Feb 24;13:1526687. doi: 10.3389/fpubh.2025.1526687 (PMC11891065; doi:10.3389/fpubh.2025.1526687)
Supplement: Supplementary file 1 [file Supplementary_file_1.docx]

# Supplementary File

**Supplementary Figures and Tables**

| **Variable** | **The question in the survey** | **Potential responses** | **Measurement** |
| --- | --- | --- | --- |
| **Employment** | What is your current employment status? | (i) Employed full-time (20 or more hours per week)  (ii) Employed part-time (less than 20 hours per week)  (iii) Unemployed looking for work  (iv) Unemployed not looking for work  (v) Retired.  (vi) Prefer not to answer | =1 if the individual is employed (i.e: either full-time or part-time) |
| **Industry** | What industry best describes your place of employment? | (i) Agriculture; (ii) Automotive Services;  (iii) Construction Services; (iv) Education;  (v) Financial Services; (vi) Food and Beverage; (v) Government agency; (vi) Healthcare sector; (vi) Manufacturing, Wholesale, and Distribution; (vii) Military; (viii) Nonprofit organization; (ix) Office; (x) Professional Services (lawyers, CPAs, consultants, etc.); (xi) Real Estate; (xii) Retail; (xiii) Tourism and Events; (xiv)Others | Dummy variables |
| **Male** | What is your sex? | (i) Male; (ii) Female; (iii) Other; (iv) Prefer not to answer | =1 if the individual is male, 0 otherwise (“Other” or “Prefer not to answer” only accounts for 0.5% of the sample) |
| **Age** | What is your year of birth? | Year of birth | Continuous variable, only focus on the group of between 18 and 65 years old. |
| **Married** | What is your civil status? | (i) Single, never married  (ii)Married or with Domestic Partner living together  (iii) Divorced or Living Separated  (iv) Widowed | = 1 if the individual is married, 0 otherwise |
| **Education** | What is your highest level of education? | (i) 5th grade or less; (ii) 6th to 8th grade; (iii) 9th to 12th grade, no diploma; (iv) High school graduate or GED completed; (iv) Some college level/Technical/Vocational degree; (v)Bachelor’s degree; (vi) Other advanced degree (Master’s, Doctoral degree) | Values of education level are between [0;1]  0 “8th grade or less”  ⅕ “9th to 12th grade, no diploma”  ⅖ “High school graduate or GED completed”  ⅗ “Some college level/Technical/Vocational degree”  ⅘ “Bachelor’s degree”  1 “Master’s, Doctoral degree” |
| **Race** | 1- What are the ethnic groups you identify with?  2- Of the ethnic groups, which one do you most identify with? | (i) African American; (ii)Caucasian; (iii)Chinese; (iv)Filipino; (v)Hawaiian; (vi)Japanese; (v) Korean; (vi) Latino; (vii) Micronesian; (viii) Native American; (ix)Portuguese; (x)Samoan; (xi) Other | Dummy variables: (1) Caucasian; (2) Hawaiian and Pacific Islanders (including Micronesian and Samoan); (3) Asian (including Chinese, Japanese, Korean, and Filipino); (4) and Others (including Native American, African American, Portuguese, and Latino) |
| **Long-COVID** | People who contracted COVID-19 often experience lingering effects from COVID-19. Have you had any lingering effects from COVID-19 that lasted more than 30 days? | (i) Yes  (ii) No | =1 if the individual chose the “Yes” option, 0 otherwise. |
| **Smoking** | During the past month, have you smoked cigarettes or chewed tobacco? | (i) More than 3 times  (ii) Once or twice  (iii) Never | =1 if the individual chose “Once or twice” or “More than 3 times”, 0 otherwise |
| **Drinking** | During the past month, have you been drunk? | (i) More than 3 times  (ii) Once or twice  (iii) Never | =1 if the individual chose “Once or twice” or “More than 3 times”, 0 otherwise |
| **Pre-existing health condition** | Do you have any of the following health conditions? (Select all that apply) | (i) Pre-diabetes; (ii) Diabetes; (iii)Immunocompromised condition; (iv) Hypertension; (v) Autoimmune disease; (vi) Cancer diagnosis and/or treatment within the past 12 months; (vii) Cardiovascular disease (CVD or heart disease); (viii) Asthma; (ix) Chronic obstructive pulmonary disease (COPD); (x) Other chronic lung disease; (xi) Sickle Cell Anemia; (xii) Depression; (xiii) Alcohol or substance use disorder; (xiv) Intravenous drug use; (xv) Other mental health disorder; (xvi) Other chronic condition (xvii) None of these | = 1 if the individual does not have any pre-existing health condition; 0 otherwise |
| **Household income per person** | Which of these categories best describes your total combined family income? (household income)  How many people are currently living in your household, including yourself? (household size) | * Household income has seven categories: (i) <$25,000; (ii)$25,000-<$50,000; (iii)$50,000-<$75,000; (iv)$75,000-<$100,000; (v)$100,000-<$150,000; (vi) Decline to respond; (vii) Don’t Know/Not sure  * Household size: (i) 1; (ii) 2; (iii) 3; (iv) 4; (v)5; (vi) 6; (vii) 7; (viii) 8; (ix)9; (x) 10 or more | Continuous variable  = household income/ household size |
| **Food insecurity**  **(includes 6 questions)** | 1. "The food that (I/we) bought just didn’t last, and (I/we) didn’t have money to get more.” Was that often, sometimes, or never true for (you/your household) in the last 12 months? *(food1)* 2. “(I/we) couldn’t afford to eat balanced meals.” Was that often, sometimes, or never true for (you/your household) in the last 12 months? *(food2)* 3. In the last 12 months, since last month, did (you/you or other adults in your household) ever cut the size of your meals or skip meals because there wasn't enough money for food? *(food3)* 4. How often did you cut the size of your meals or skip meals because there wasn't enough money for food? *(food4)* 5. In the last 12 months, did (you/you or other adults in your household) ever eat less than you felt you should because there wasn't enough money for food? *(food5)* 6. In the last 12 months, were you ever hungry but didn't eat because there wasn't enough money for food? *(food6)* | Question (1) and (2) have the potential responses: (i) Often true; (ii) Sometimes true; (iii)Never true; (iv) Don't know or Refused  Question (3) has the potential responses : (i)Yes; (ii) No  Question (4) has the potential responses: (i) Almost every month  (ii) Some months but not every month; (iii) Only 1 or 2 months; (iv) Don’t know  Question (5) and (6) have the potential responses: (i)Yes; (ii) No; (iii) Don’t know | Food1 = 1 if "Sometimes true" or "Often true"; =0 if "Never true"  Food2 = 1 if "Sometimes true" or "Often true"; =0 if "Never true"  Food3= 1 if "Yes", =0 if "No"  Food4 = 1 if "Almost every month" or "Some months but not every month"; = 0 if 'Only 1 or 2 months"  Food5 = 1 if "Yes"; =0 if "No"  Food6 = 1 if "Yes"; =0 if "No"  Total score = Food1 + Food2 + Food3 +Food4 + Food5 + Food6 |
| **Official trust** | How much do you trust each of these sources to provide correct information?  -Your doctor or health care provider (trust1)  -News on the radio, TV, online, or in newspapers(trust2)  -U.S. government (trust3)  -U.S. Center for Disease Control (trust4) | (i) “Not at all”; (ii) “Don’t know”; (iii) “ A little”; (iv) “Somewhat”; (v) “A great deal” | Score of each response: 0: “Not at all”;  1: “Don’t know”;  2: “ A little”;  3: “Somewhat”;  4: “A great deal”.  Official trust score =  $\frac{trust1 + trust2 + trust3 +trust4}{16}$  Trust score is between 0 and 1 |
| **Unofficial trust** | How much do you trust each of these sources to provide correct information?  -Your faith leader (trust5)  -Your close friends and members of your family (trust6)  -People you go to work or class with or other people you know (trust7)  -Your contacts on social media (trust8) | (i) “Not at all”; (ii) “Don’t know”; (iii) “ A little”; (iv) “Somewhat”; (v) “A great deal” | Score of each response: 0: “Not at all”;  1: “Don’t know”;  2: “ A little”;  3: “Somewhat”;  4: “A great deal”.  Official trust score =  $\frac{trust5 + trust6 + trust7 +trust8}{16}$  Trust score is between 0 and 1 |
| **Safety against COVID-19** | Do you feel safe in your neighborhood/community against COVID-19? | (i) Very safe; (ii) Safe; (iii) Neutral; (iv) Unsafe; (v) Very unsafe | =1 if the individual chose “Very safe” or “Safe” |
| **Dependent Variable** |  |  |  |
| **Self-esteem** | Below is a list of statements dealing with your general feelings about yourself. Please select the answer that best reflects your feeling.  -I feel that I'm a person of worth, at least on an equal plane with others. (self1)  -I feel that I have a number of good qualities. (self2)  -All in all, I am inclined to feel that I am a failure. (self3)  -I am able to do things as well as most other people. (self4)  -I feel I do not have much to be proud of. (self5)  -I take a positive attitude toward myself. (self6)  -On the whole, I am satisfied with myself. (self7)  -I wish I could have more respect for myself. (self8)  -I certainly feel useless at times. (self9)  -At times I think I am not good at all. (self10) | (i) Strongly agree  (ii) Agree  (iii) Disagree  (iv) Strongly disagree | Mark of each option:  0- Strongly Disagree  1- Disagree  2- Agree  3- Strongly agree  Total Self-esteem score = self1+ self2+ self3+ self4 +self5+ self6+ self7+ self8+ self9 + self10  Value of total self-esteem score is between 0 and 30 |
| **Depression** | Below is a list of some of the ways you may have felt or behaved. Please indicate how often you have felt this way during the past week by checking the appropriate box for each row. (Select one option per row)  -I was bothered by things that usually don't bother me (dep1)  -I had trouble keeping my mind on what I was doing (dep2)  -I felt depressed (dep3)  -I felt that everything I did was an effort (dep4)  -I felt hopeful about the future (dep5)  -I felt fearful (dep6)  -My sleep was restless (dep7)  -I was happy (dep8)  -I felt lonely (dep9)  -I could not "get going" (dep10) | (i) Rarely or none of the time (less than 1 day)  (ii) Some or a little of the time (1‐2 days)  (iii) Occasionally or a moderate amount of time (3‐4 days)  (iv) All of the time (5‐7 days) | Mark of each option:  0- Rarely or none of the time (less than 1 day)  1- Some or a little of the time (1‐2 days)  2- Occasionally or a moderate amount of time (3‐4 days)  3- All of the time (5‐7 days)  Total depression score = dep1+ dep2+ dep3+ dep4 + dep5+ dep6+ dep7+ dep8+ dep9 + dep10  Value of total self-esteem score is between 0 and 30 |
| **Suicidal Ideation** | During the past year, did you ever seriously consider attempting suicide? | (i) Yes  (ii) No | =1 if the individual chose “Yes” |

**Table A1:** Variable Definitions and Scoring

|  | **Depression** | **Low Self-esteem** | **Suicidal ideation** |
| --- | --- | --- | --- |
| Employment | -0.028*  (0.012) | -0.068***  (0.007) | -0.025***  (0.003) |
| Male | -0.054***  (0.010) | -0.019  (0.012) | 0.015  (0.010) |
| Age | -0.001  (0.001) | -0.003***  (0.000) | -0.002***  (0.000) |
| Married | -0.088***  (0.008) | -0.096***  (0.018) | -0.029***  (0.006) |
| Education | 0.061  (0.044) | 0.009  (0.050) | 0.023  (0.019) |
| Caucasian | 0.020  (0.026) | -0.027  (0.028) | 0.018***  (0.005) |
| NHPI | -0.068**  (0.026) | -0.079*  (0.037) | -0.001  (0.014) |
| Asian | -0.086***  (0.023) | -0.033**  (0.011) | 0.002  (0.006) |
| Long-COVID | 0.087**  (0.034) | -0.002  (0.020) | 0.013  (0.014) |
| Pre-existing health condition | -0.128***  (0.019) | -0.087***  (0.005) | -0.048***  (0.005) |
| Smoking | 0.051**  (0.016) | 0.052**  (0.017) | 0.018*  (0.007) |
| Drinking | 0.043**  (0.016) | 0.025  (0.036) | -0.004  (0.008) |
| Household income per person | 0.019  (0.038) | -0.062  (0.025) | -0.006  (0.005) |
| Food insecurity | 0.209***  (0.015) | 0.074***  (0.006) | 0.034***  (0.007) |
| Official trust | 0.031  (0.034) | -0.074***  (0.010) | -0.030  (0.019) |
| Unofficial trust | -0.074*  (0.037) | -0.076***  (0.011) | -0.045***  (0.007) |
| Safety against COVID-19 | -0.099***  (0.015) | -0.027*  (0.013) | -0.011*  (0.005) |
| N | 2270 | 2226 | 2174 |

**Note:** Statistical significance level: ∗p < 0.05, ∗∗ p < 0.01, ∗∗∗p < 0.001. Robust standard errors clustered at county level and reported in the parentheses. Each regression also includes industry, county and time of collection fixed effects.

**Table A2: Marginal effect of social determinants on mental health**


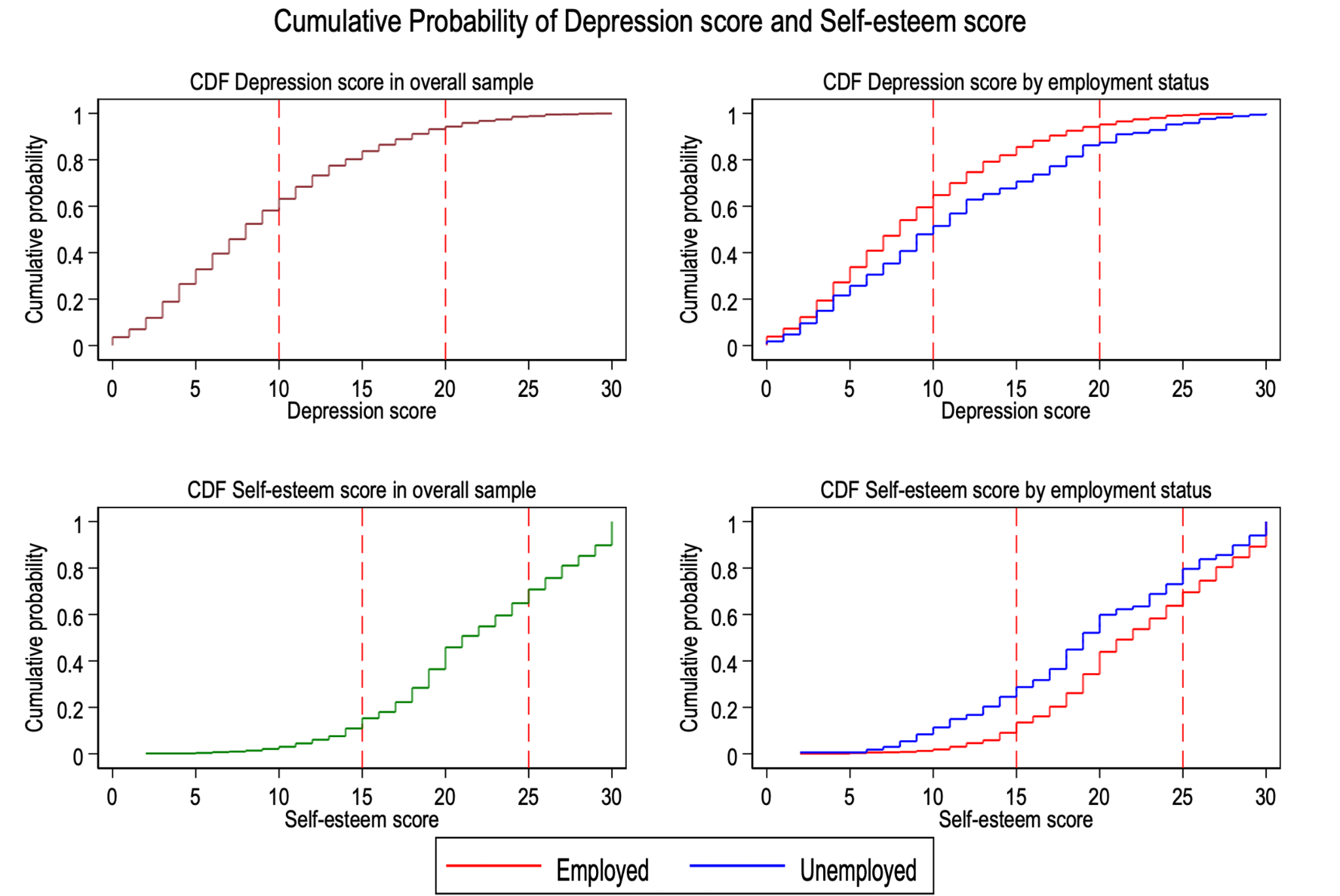


**Figure A1:** Data of people aged between 18 and 65 that was collected in the baseline (May, 2022) (87.92% of the sample are employed and 12.08% of them are unemployed). Depression score is calculated based on the CES-D 10 item scale with two cutoffs at 10 and 20 points (two red vertical lines). Self-esteem score is calculated based on the Rosenberg Self- esteem 10-item scale with two cutoffs at 15 and 25 points (two red vertical lines). Cumulative probability of depression score of the employed people is higher than the unemployed people. By contrast, the cumulative probability of self-esteem score of the employed people is lower than the unemployed people. These cumulative probabilities suggest that the employed people are less likely to experience depression, and more likely to have high self-esteem.

**
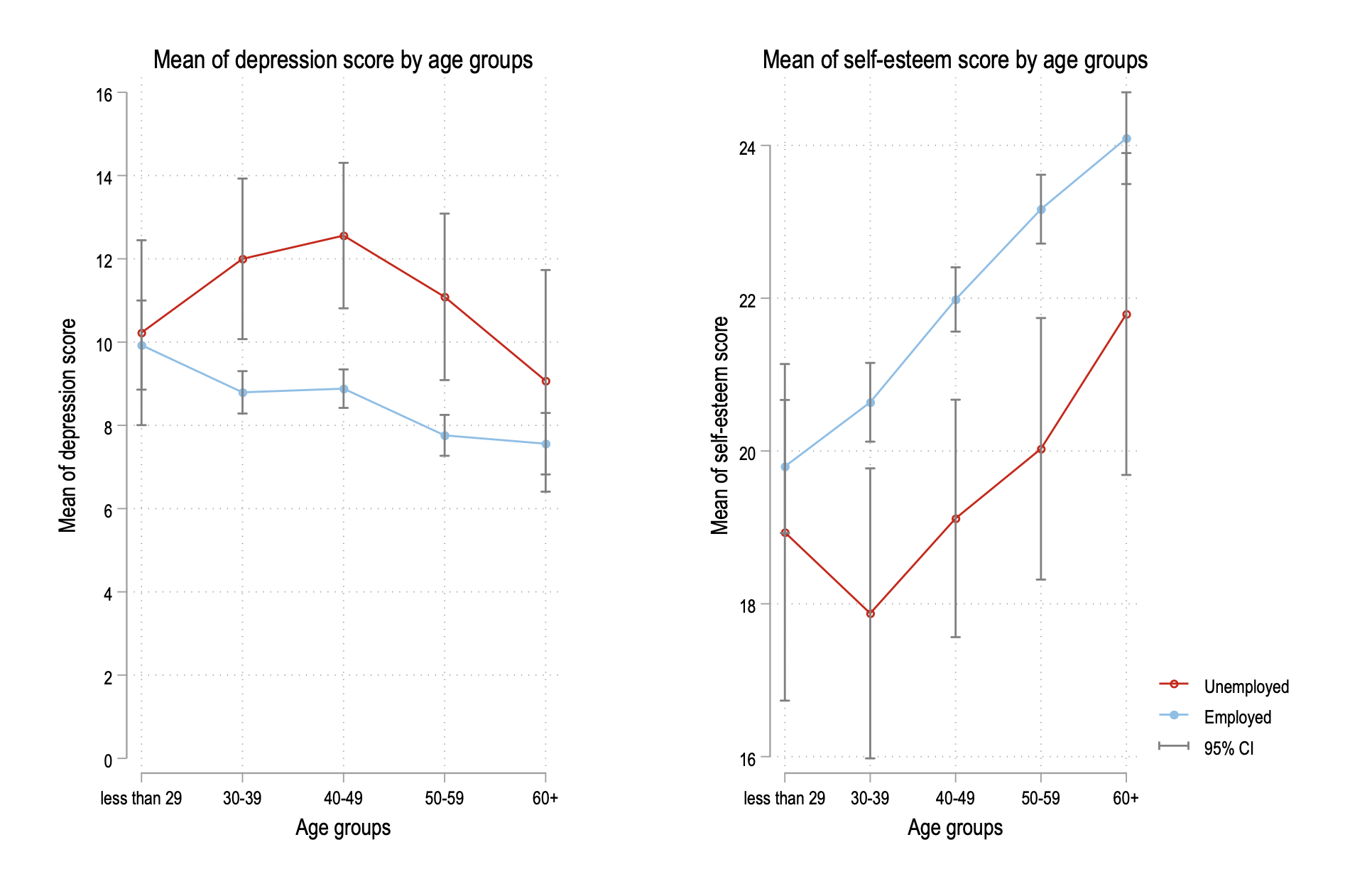
Figure A2:** Mean of depression score and self-esteem score by age groups.


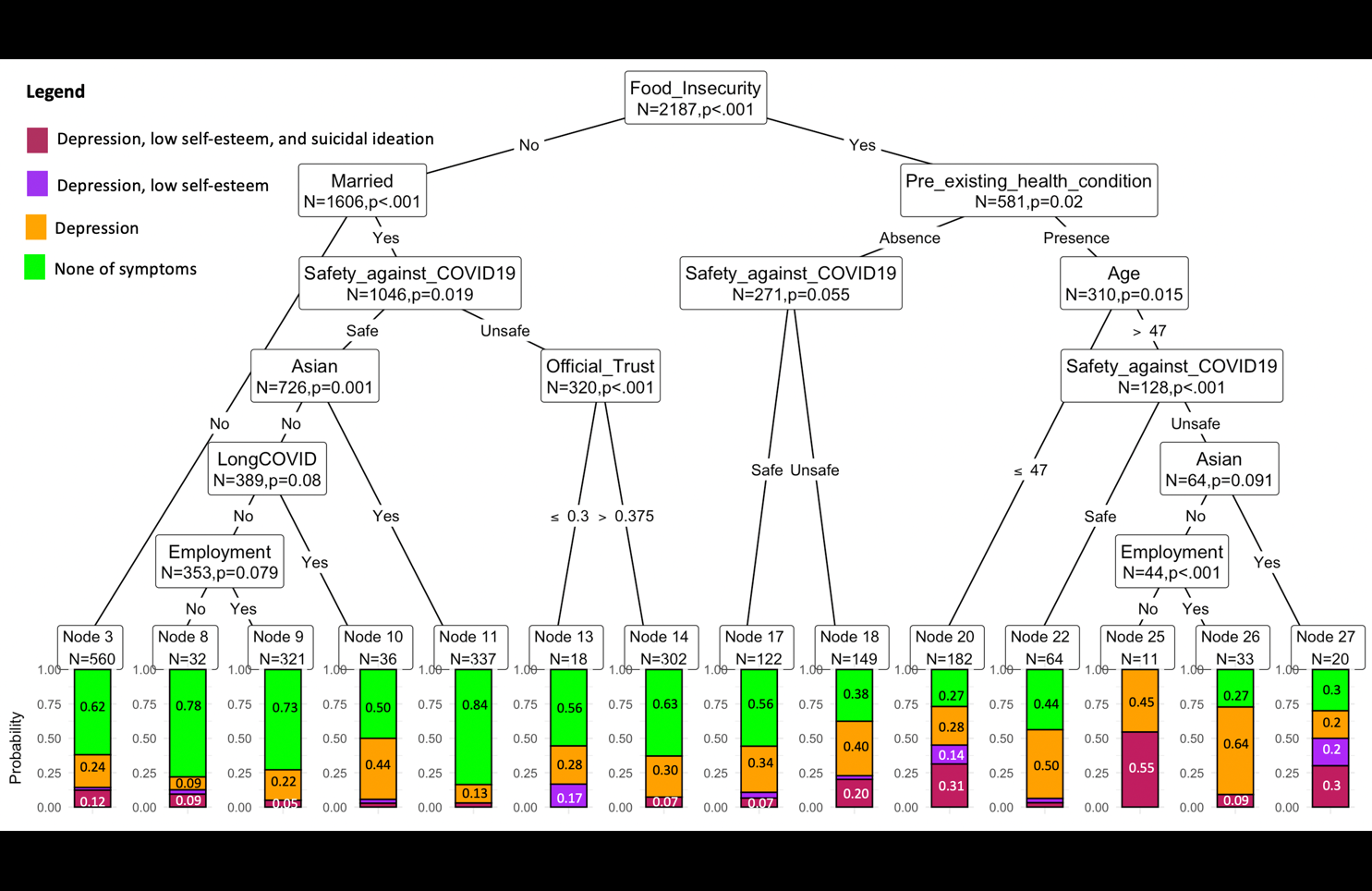
**Figure 3A1**: The graph depicts the decision tree for classifying the number of mental health issues with a statistical significance level of 1%.


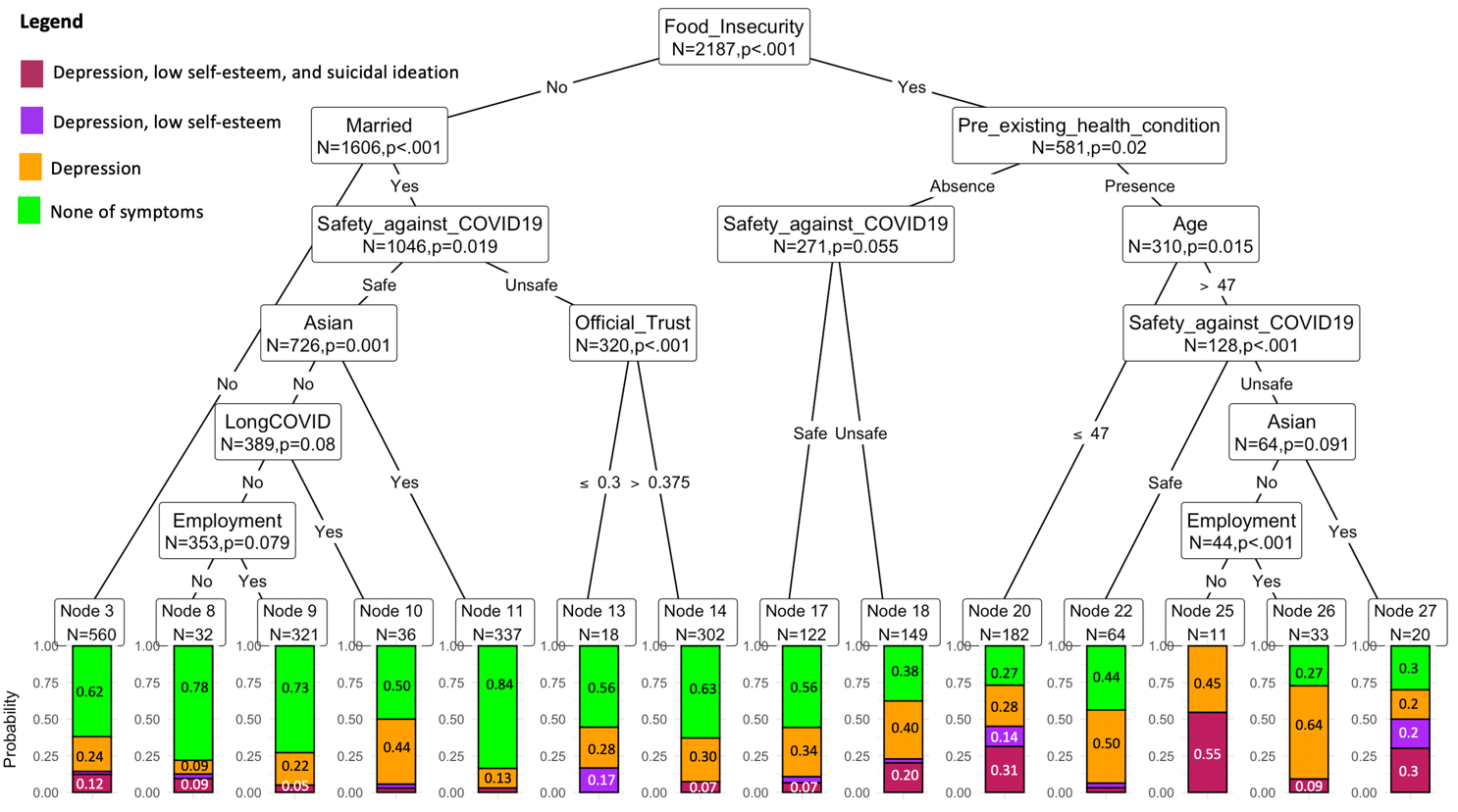
**Figure 3A2**: The graph depicts the decision tree for classifying the number of mental health issues with a statistical significance level of 10%.
